# Microglia Gone Rogue: Impacts on Psychiatric Disorders across the Lifespan

**DOI:** 10.3389/fnmol.2017.00421

**Published:** 2018-01-04

**Authors:** Tuan Leng Tay, Catherine Béchade, Ivana D’Andrea, Marie-Kim St-Pierre, Mathilde S. Henry, Anne Roumier, Marie-Eve Tremblay

**Affiliations:** ^1^Institute of Neuropathology, University of Freiburg, Freiburg, Germany; ^2^Faculty of Biology, University of Freiburg, Freiburg, Germany; ^3^INSERM UMR-S 839, Paris, France; ^4^Sorbonne Universités, Université Pierre et Marie Curie (UPMC), Paris, France; ^5^Institut du Fer à Moulin, Paris, France; ^6^Axe Neurosciences, CRCHU de Québec—Université Laval, Québec, QC, Canada; ^7^Département de Médecine Moléculaire, Université Laval, Québec, QC, Canada

**Keywords:** microglia, early-life stress, microgliopathies, autism spectrum disorder, major depressive disorder, schizophrenia, aging, neurodegenerative disease

## Abstract

Microglia are the predominant immune response cells and professional phagocytes of the central nervous system (CNS) that have been shown to be important for brain development and homeostasis. These cells present a broad spectrum of phenotypes across stages of the lifespan and especially in CNS diseases. Their prevalence in all neurological pathologies makes it pertinent to reexamine their distinct roles during steady-state and disease conditions. A major question in the field is determining whether the clustering and phenotypical transformation of microglial cells are leading causes of pathogenesis, or potentially neuroprotective responses to the onset of disease. The recent explosive growth in our understanding of the origin and homeostasis of microglia, uncovering their roles in shaping of the neural circuitry and synaptic plasticity, allows us to discuss their emerging functions in the contexts of cognitive control and psychiatric disorders. The distinct mesodermal origin and genetic signature of microglia in contrast to other neuroglial cells also make them an interesting target for the development of therapeutics. Here, we review the physiological roles of microglia, their contribution to the effects of environmental risk factors (e.g., maternal infection, early-life stress, dietary imbalance), and their impact on psychiatric disorders initiated during development (e.g., Nasu-Hakola disease (NHD), hereditary diffuse leukoencephaly with spheroids, Rett syndrome, autism spectrum disorders (ASDs), and obsessive-compulsive disorder (OCD)) or adulthood (e.g., alcohol and drug abuse, major depressive disorder (MDD), bipolar disorder (BD), schizophrenia, eating disorders and sleep disorders). Furthermore, we discuss the changes in microglial functions in the context of cognitive aging, and review their implication in neurodegenerative diseases of the aged adult (e.g., Alzheimer’s and Parkinson’s). Taking into account the recent identification of microglia-specific markers, and the availability of compounds that target these cells selectively *in vivo*, we consider the prospect of disease intervention via the microglial route.

## Introduction

The modulation of higher order cognitive functions and the dysregulation thereof that leads to neuropsychiatric diseases may commonly be attributed to brain wiring and neural connectivity. Nevertheless, mounting evidence that non-neural microglia play critical and specific roles during brain development, homeostasis and plasticity, with consequences on neurodevelopmental and neuropsychiatric disorders, should be strongly considered in this context (reviewed in Prinz and Priller, 2014; Tay et al., 2017b). Microglia are tissue resident macrophages of the central nervous system (CNS) parenchyma that share the same yolk sac origin as other long-living tissue macrophages (Gomez Perdiguero et al., 2015). Thus the myeloid identity of microglia makes this population unique within the CNS, as they could be strong candidates for therapeutic interventions, without direct impact on cell types of the neuroectodermal lineage within the brain. Previously we examined in detail the growing literature on the varied roles exerted by microglial cells in the healthy brain, across the lifespan, during which they are constant surveillants, and not simply orchestrators of immune responses (reviewed in Tremblay, 2011; Tremblay et al., 2011; Tay et al., 2017b). Here we expand the discussion and focus on the impact of defective microglial physiological roles, from prenatal to aged CNS, on the emergence of various neurodevelopmental, neuropsychiatric and neurodegenerative disorders, and discuss the potential for treatment by specifically targeting microglial cells.

## Establishment and Maintenance of CNS Microglia

Even when considering the microglia distinct from other CNS cell types, it is important to recognize their unifying characteristics as much as their inherent differences. The mesodermal microglial network begins to establish itself at 9.0 days post conception in the murine CNS, prior to the appearance of the neuroectodermal lineage (reviewed in Tay et al., 2016, 2017b). Several studies support the notion that yolk sac-derived endogenous microglia of the brain parenchyma are a self-maintaining population that persists and functions throughout the animal’s lifespan (Alliot et al., 1999; Ajami et al., 2007; Ginhoux et al., 2010; Hashimoto et al., 2013; Hoeffel et al., 2015). Yet, recent lineage tracing studies that were conducted in mouse or in human, using genetic approaches, integration of thymidine analogs (Askew et al., 2017; Tay et al., 2017a) or carbon dating (Réu et al., 2017), have provided further evidence that microglial lifespan varies across brain compartments (Lawson et al., 1992). The significance of the varied turnover kinetics of microglia on their brain microenvironment is currently unclear. While this myeloid population purportedly originates from a single erythromyeloid progenitor (Ginhoux et al., 2010; Gomez Perdiguero et al., 2015), microglial heterogeneity is reflected in their varied distribution and morphology within the CNS (Lawson et al., 1990; De Biase et al., 2017), alongside brain region-dependent differences in gene expression (Doorn et al., 2015), bioenergetics, and immunophenotype (Grabert et al., 2016). Variations in microglial density between male and female parietal cortex, amygdala, hippocampus, and preoptic area (Schwarz et al., 2012; Lenz et al., 2013), and sex differences in microglial response to neuropathic pain (Sorge et al., 2015), have been reported in mice. Groundbreaking studies also proposed that the microenvironment in which microglia evolve influences their tissue-specific identities due to a selection pressure for exclusive gene enhancers (Gosselin et al., 2014, 2017; Lavin et al., 2014). Nonetheless, we are still in a conundrum as microglia have, until now, mostly been investigated as a single entity as compared to other cells of myeloid origin (Hickman et al., 2008; Butovsky et al., 2012, 2014; Gautier et al., 2012; Chiu et al., 2013).

What are the factors required for the establishment and maintenance of microglia? We reviewed this in detail previously (Tay et al., 2017b). Here we discuss the new players reported during this past year and briefly highlight the key transcription factors and signaling pathways that are particularly significant to the associated pathologies covered below. Signaling via the microglial colony-stimulating factor 1 receptor (CSF1R; Ginhoux et al., 2010; Erblich et al., 2011; Elmore et al., 2014) in particular via the alternative CSF1R ligand interleukin (IL)-34, was reported to be necessary for the survival and proliferation of microglia throughout early to adult stages (Greter et al., 2012; Wang et al., 2012). In various contexts, the purinergic ionotropic receptor P2X7 (Rigato et al., 2012), and cytokine transforming growth factor β (TGFβ; Butovsky et al., 2014) were described to regulate microglial cell density and maturation. The recruitment of microglia into CNS compartments where they provide essential support during development requires fractalkine (CX3CL1/CX3CR1) signaling (Maggi et al., 2011; Paolicelli et al., 2011; Rogers et al., 2011; Hoshiko et al., 2012; Ueno et al., 2013; Zhan et al., 2014; Pagani et al., 2015; Hellwig et al., 2016; Milior et al., 2016) and neurogenesis-dependent CXCL12/CXCR4 signaling (Arnò et al., 2014). More recent studies also unveiled the importance of transcription factors such as MAFB (Matcovitch-Natan et al., 2016) and Sal-like 1 (SALL1; Buttgereit et al., 2016; Koso et al., 2016) for maintenance of adult microglial homeostasis and function. Besides the transmembrane protein 119 (TMEM119), a microglia-specific cell surface protein of unknown function expressed from early postnatal development until adulthood (Bennett et al., 2016), *Sall1* was proposed to constitute a microglial signature gene considering its lack of expression in other mononuclear phagocytes and CNS cell types (Buttgereit et al., 2016). Regulating the phagocytic functions of adult microglia, the TAM receptor tyrosine kinases MER and AXL were described to be necessary for the removal of apoptotic cells resulting from adult neurogenesis (Fourgeaud et al., 2016). Microglia lacking TAM were shown to be less motile *in vivo* with delayed response to brain damage, thus underscoring the importance of MER and AXL in modulating microglial physiology (Fourgeaud et al., 2016). From a systematic analysis of the transcriptional regulation and epigenetic signature of microglia from yolk sac to adult stages, three distinct temporal stages of microglial development, namely the early-microglia, pre-microglia and adult microglia, were unveiled. The authors further demonstrated that the microglial developmental program is sensitive to environmental perturbations such as prenatal immune activation and microbiome alteration (Matcovitch-Natan et al., 2016). Indeed, it was shown earlier that reconstitution of the gut of mice raised in a germ-free facility with short-chain fatty acid by-products of bacterial fermentation was sufficient to recover a normal ramified microglial phenotype (Erny et al., 2015).

## Physiological Functions of Microglia in the Brain

Microglia fulfill their roles during development, homeostasis and plasticity mainly through their sensing and scavenging activities, and secretion of trophic factors, cytokines and chemokines. The physiological functions of microglia at steady-state, previously discussed at length (Tay et al., 2017b), are summarized below to provide a context for our main discussions on the impact of defective microglia on psychiatric disorders.

In CNS development, microglia regulate the turnover of neural precursors and neurons by phagocytosis of apoptotic cells and excess newborn neurons (Marín-Teva et al., 2004; Peri and Nüsslein-Volhard, 2008; Swinnen et al., 2013). Furthermore, microglia support neurogenesis, neuronal survival, and the maintenance and maturation of oligodendrocyte progenitor cells through their release of trophic cytokines, also in the adult brain (Sierra et al., 2010; Arnò et al., 2014; Hagemeyer et al., 2017; Wlodarczyk et al., 2017). The positioning of microglial cells along axonal tracts suggests a role in neuronal wiring during embryonic and postnatal stages (Cho et al., 2013; Squarzoni et al., 2014). From early postnatal development until normal aging, a main contribution of microglia in the healthy brain is their activity-based regulation of neuronal activity and synaptic plasticity, which is notably exerted through the refinement of synaptic connections (Wake et al., 2009; Tremblay et al., 2010; Bialas and Stevens, 2013). Real-time two-photon *in vivo* imaging has provided convincing evidence that microglia are extremely dynamic cells. Surveillant microglia continuously extend and retract highly motile processes to interact with their microenvironment, including synapses, at all stages of life (Davalos et al., 2005; Nimmerjahn et al., 2005; Wake et al., 2009; Tremblay et al., 2010; Li et al., 2012). Microglia-synapse interactions regulate the formation and elimination of synapses. As professional phagocytes of the CNS, microglia engulf axon fragments and terminals, as well as dendritic spines, thereby contributing to a crucial pruning function that is regulated by neuronal activity, learning and memory, and the ongoing experience (Watts et al., 2004; Tremblay et al., 2010; Paolicelli et al., 2011; Schafer et al., 2012; Bialas and Stevens, 2013; Squarzoni et al., 2014). Activity- or learning-based dendritic spine formation (Parkhurst et al., 2013; Miyamoto et al., 2016) is mediated through microglial release of brain-derived neurotrophic factor (BDNF; Parkhurst et al., 2013), and their elimination of axon terminals by a TGFβ-dependent cascade that involves the complement proteins C1q and C3 tagging synapses for microglial complement receptor 3 (CR3)-mediated removal (Schafer et al., 2012; Bialas and Stevens, 2013). Fractalkine signaling is also required for hippocampal-associated learning and memory, and the adaptation to a stressful or enriched environment (Maggi et al., 2011; Rogers et al., 2011; Milior et al., 2016). Taken together, functional microglia are essential for synaptic formation, maintenance and plasticity, as well as remodeling of neural networks in response to learning and environmental challenges.

## Contribution of Microglia to the Environmental Risk Factors for Psychiatric Diseases

**Figure 1:**
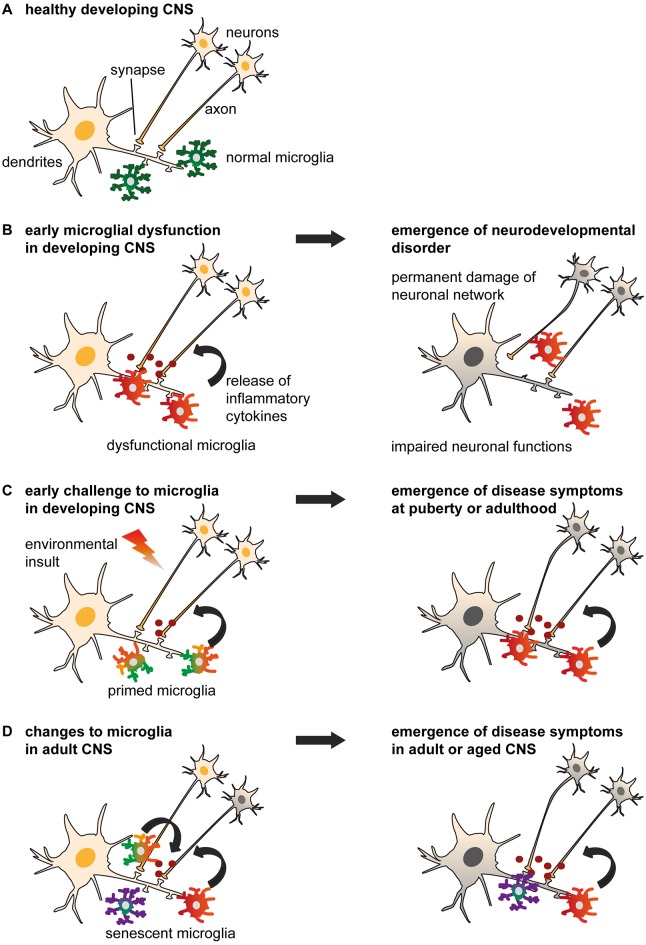
Models of non-physiological microglia and their impacts on the onset of disease. **(A)** Normal microglia-neuron interactions in the central nervous system (CNS). **(B)** An early microglial dysfunction due to genetic or environmental (e.g., maternal or perinatal stress, inflammation, dietary deficiency) risk factors can lead to impaired neuronal functions and an early emergence of neurodevelopmental disorders. Aberrant release of cytokines, impaired pruning and phagocytic activities can affect neuronal densities, maturation and wiring, thus translating into permanent defects of the neural network. These include an imbalance of excitability to inhibition, or altered connectivity between brain regions, which are sufficient to induce the onset of psychiatric disorders during childhood (e.g., Nasu-Hakola disease (NHD), hereditary diffuse leukoencephaly with spheroids (HDLS), Rett syndrome (RTT), autism spectrum disorder (ASD) and obsessive-compulsive disorder (OCD)), or render an individual vulnerable to subsequent insults. **(C)** An early environmental challenge can prime microglia by altering their maturation and inflammatory states with limited immediate impacts on the neuronal network, thus resulting in asymptomatic changes. However, primed microglia are rendered more susceptible to subsequent challenges such as stress or chronic infections, and may adopt abnormal patterns of cytokine secretion or synaptic pruning later in life. These changes may progressively damage the neural system during puberty and adulthood, leading to the emergence of psychiatric disorders (e.g., alcohol and drug abuse, major depressive disorder (MDD), schizophrenia, bipolar disorder (BD), eating disorders and sleep disorders). **(D)** Changes to microglial phenotypes occurring during adulthood may be accelerated by genetic or environmental factors. Non-physiological microglia may have reduced capability to restore CNS homeostasis, or contribute to neurodegeneration and altered wiring, which result in the onset of cognitive disorders in adult [as in **(C)**] and aged patients (e.g., Alzheimer’s disease (AD), dementia and Parkinson’s disease (PD)).

Enduring fevers or maternal infections during pregnancy, and physiological injuries at birth (e.g., infection, hypoxia-ischemia and trauma) increase the risk for autism, attention deficit and hyperactivity disorder, and schizophrenia (Patterson, 2009; Brown and Derkits, 2010; Knuesel et al., 2014; Hagberg et al., 2015; Hornig et al., 2017; Instanes et al., 2017). Childhood maltreatment that comprises physical or emotional neglect and sexual abuse, is considered a major risk factor for adult psychiatric conditions that include eating disorders, alcohol and drug abuse, as well as depression (Kessler et al., 2010; Scott et al., 2012). Maternal immune activation is considered a “neurodevelopmental disease primer” that combines with genetics and other environmental cues to induce mental disorders (reviewed in Knuesel et al., 2014; Meyer, 2014) Similarly, stressful events during adulthood, or chronic post-traumatic disorder, increase the risk for depression (Kessler, 1997), accelerate aging, and may favor neurodegenerative disorders, including the sporadic, late onset, forms of Alzheimer’s disease (AD) and Parkinson’s disease (PD; Fidler et al., 2011; Miller and Sadeh, 2014). Such challenges could increase the vulnerability to psychiatric disorders by disrupting microglial functions. Microglial “priming” or increased sensitivity to subsequent insults is one of the proposed mechanisms. Primed microglia differ from reactive cells (frequently referred to as “activated microglia” in literature), by displaying increased expression of genes related to phagocytosis, proliferation, and vesicular release (Orre et al., 2014; Holtman et al., 2015). The response to inflammatory challenges is exacerbated in primed microglia, which release increasing amounts of cytokines (Norden et al., 2015). Priming could also prevent microglia from exerting their normal physiological functions, directly impairing neurogenesis, synaptogenesis, and the wiring of brain circuits, with severe impacts on learning, memory and other cognitive processes (Figure 1).

As sentinels, microglia are likely the first CNS cell type to sense psychological stress and peripheral inflammation, and mediate the effects of perinatal challenges on the developing brain. Offspring exposed to lipopolysaccharide (LPS; from gram-negative bacteria) during embryonic development show mispositioned cortical interneurons at postnatal stages (Squarzoni et al., 2014) and altered glutamatergic transmission as well as long-term potentiation (LTP) in adolescence (Roumier et al., 2008). Animals challenged by inflammation during pre- or post-natal development, or maternal separation, exhibit long-lasting microglial alterations, including an increased prevalence of ameboid morphologies (reviewed in Boksa, 2010; Johnson and Kaffman, 2017). Early-life stress and prenatal inflammation also induce changes in microglial molecular signature (*C1q* and *Cx3cr1*) and phagocytic activity *ex vivo* (Delpech et al., 2016; Mattei et al., 2017), but their effects on phagocytosis are opposite, with prenatal inflammation being inhibitory (Mattei et al., 2017). Prenatal inflammation accelerates the transcriptomic maturation profile of early postnatal microglia towards an adult signature (Matcovitch-Natan et al., 2016). This shift may restrict microglial physiological functions at crucial stages of development, leading to connectivity alterations or excitatory/inhibitory synapses imbalance, and associated behavioral deficits. Maternal or perinatal stress or immune challenge in rodents, induced by cytokines or surrogates of bacteria (LPS) or viruses (viral RNA mimic polyinosinic-polycytidylic acid; Poly I:C), result in behavioral defects at adolescence or adulthood. These comprise anxiety, impairment of memory, sociability and sensorimotor gating, increased repetitive behavior and enhanced psychostimulants sensitivity (reviewed in Weinstock, 2001; Meyer and Feldon, 2009; Boksa, 2010; Careaga et al., 2017). In the offspring exposed to maternal immune challenge, abnormalities in dopaminergic and GABAergic systems, including increased dopaminergic afferences in the nucleus accumbens (NAc) and decreased inhibition of parvalbumin-positive interneurons on cortical pyramidal neurons were also reported (reviewed in Meyer and Feldon, 2009; Estes and McAllister, 2016).

In addition to stress and infections, epidemiological studies on n-3 poly-unsaturated fatty acids (PUFA), contained mainly in seafood and fishes, but not produced by humans, support the belief that a well-balanced diet is essential. An n-3 PUFA-rich maternal diet was shown to improve the intelligence quotient of children (Helland et al., 2008), whereas the absence of dietary n-3 PUFA negatively impacted on the intellectual performances (Hibbeln et al., 2007; reviewed in Luchtman et al., 2013). In mice, an n-3 PUFA-deficient diet during gestation induced the deregulation of hippocampal *Egr1*, *c-Jun, Bdnf* associated with neuronal plasticity in the adolescent offspring. These deleterious effects correlated with impaired microglial motility (in *Cx3cr1-GFP* reporter mice) and decreased expression of inflammation-associated genes *ex vivo*, without alteration in IBA1-positive microglial number (Madore et al., 2014). Further investigation is warranted to determine whether these effects resulted from the anti-inflammatory properties of n3-PUFA acting on microglia, or indirectly from changes in the gut microbiota (Madore et al., 2016). Microglia are affected by other nutritional factors. For instance, a high-fat and high-sucrose maternal diet that induces gestational diabetes in rats, a condition that is associated with decreased cognitive performance and psychiatric disorders in humans, increased the protein levels of pro-inflammatory cytokines and the prevalence of ameboid IBA1-positive microglia in rat neonates (Vuong et al., 2017). Such diet also impaired object memory and induced a shift of hippocampal microglia toward less ramified morphologies in the adult offspring (Vuong et al., 2017). At adulthood, high-fat diet reduces spine density in the dentate gyrus and prefrontal cortex, in addition to impairing novel object memory (Bocarsly et al., 2015; Hao et al., 2016). This diet enhanced the length of IBA1-positive microglial processes (Bocarsly et al., 2015) and the phagocytic activity of isolated microglia toward synaptosomes (Hao et al., 2016), which could mediate the induced changes in connectivity. The saturated fatty acids, which are overrepresented in high-fat, Western-type diet, and which accumulate in the hypothalamus, exert direct pro-inflammatory effects on microglia (identified by IBA1, CD11b or CD68 staining), both in culture and *in vivo* (Valdearcos et al., 2014).

## Roles of Microglia in Neurodevelopmental Disorders

**Figure 2:**
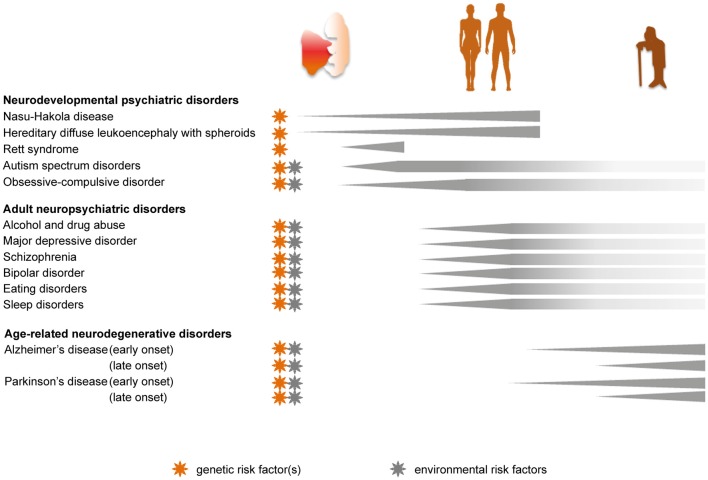
Genetic and environmental implications for the role of microglia in the progression of psychiatric diseases across the lifespan. Pathologies caused by, or linked to, genetic risk factors are marked with an orange star. Disease-associated priming of microglia due to environmental risk factors, such as perinatal inflammation, maternal and early life stress, dietary imbalance and chronic stress during adulthood, is indicated with a gray star.

Abnormal wiring of brain circuits during development is proposed to underlie mood instability, abnormal behavior, and cognitive defects that may arise and develop later in life. Here we review the evidence that certain psychiatric diseases with a neurodevelopmental origin are linked to an early microglial dysfunction (Figures 1, 2).

Nasu-Hakola disease (NHD) and *hereditary diffuse leukoencephaly with spheroids* (HDLS) are human diseases associated with behavioral and cognitive alterations resulting from mutations in genes expressed by microglia. These diseases, termed “microgliopathies” (Rademakers et al., 2011), can be considered neurodevelopmental as the microglial mutations affect brain development from prenatal stages, even though disease onset occurs around the fourth decade of life.

NHD is a rare autosomal microgliopathy with a psychiatric outcome, characterized by presenile dementia and bone cysts resulting in premature death (Hakola, 1972; Nasu et al., 1973). The most characteristic abnormalities observed in the postmortem brain of NHD patients comprise a marked loss of myelin, the presence of axon spheroids, astrogliosis, and CD68-positive cells with a large soma and few processes, named “activated microglia”, in the white matter of frontal and temporal lobes (Paloneva et al., 2001). NHD is caused by recessive gene mutations of *DAP12* (*TYRO Protein Tyrosine Kinase Binding Protein*) or *TREM2* (*Triggering Receptor Expressed On Myeloid Cells 2*, Paloneva et al., 2000, 2002; Bianchin et al., 2004). DAP12 is a transmembrane protein that transduces signals from several lymphoid and myeloid receptors including TREM2. In mice, *Trem2* and *Tyrobp* (the gene encoding DAP12) are expressed by a variety of innate immune cells (Lanier and Bakker, 2000), such as microglia in the CNS (Roumier et al., 2004; Wakselman et al., 2008; Hsieh et al., 2009; Hickman et al., 2013). The molecular mechanisms linking the TREM2-DAP12 pathway to NHD remain elusive. However, analysis of DAP12-deficient mice revealed several neuronal alterations at adulthood, notably enhanced hippocampal LTP (Roumier et al., 2004) and impaired sensorimotor gating (Kaifu et al., 2003), which could account for the cognitive and behavioral symptoms of NHD patients. Transcriptional profiling of DAP12-deficient microglia at embryonic day 17.5 revealed a down-regulation of genes involved in neurite formation accompanied by defasciculation of corpus callosum axons (Pont-Lezica et al., 2014), while the lack of DAP12 impaired the outgrowth of dopaminergic axons and altered the positioning of neocortical interneurons in prenatal mice (Squarzoni et al., 2014). A role for TREM2-DAP12 pathway in clearing apoptotic neurons was also demonstrated in microglial culture (Takahashi et al., 2005; Wakselman et al., 2008) and during developmental cell death *in vivo* in mice (Takahashi et al., 2005; Wakselman et al., 2008). These data show that prenatal dysfunction of microglia due to compromised TREM2-DAP12 signaling can affect synaptic function as well as axonal outgrowth and guidance, which may trigger psychiatric defects in humans.

Another microgliopathy is HDLS, a rare autosomal dominant disease defined by progressive motor, behavioral, and cognitive alterations leading to severe dementia (Axelsson et al., 1984). Studies have shown that HDLS patients present degenerative alterations reminiscent of NHD (Axelsson et al., 1984; Rademakers et al., 2011; Sundal et al., 2012; Konno et al., 2014). HDLS is caused by mutations in the tyrosine kinase domain of *CSF1R* (Rademakers et al., 2011). In mouse brain, *Csf1r* is expressed by microglia (Geissmann et al., 2010) and, as mentioned above, is essential to their development and maintenance. The mechanisms linking CSF1R dysfunction to HDLS remain unknown. However, DAP12 regulates the ability of CSF1R to control the survival and proliferation of bone marrow-derived macrophages *in vitro* (Otero et al., 2009). This suggests that NHD and HDLS may involve a deficit of the same signaling pathway, induced through their respective mutations of *TYROBP*, *TREM2* or *CSF1R* genes.

*Rett syndrome* (RTT) is a X-linked mental disorder affecting mostly girls, with an onset in the first 2 years of life, in which multiple neurologic, motor, digestive and respiratory symptoms combine with an intellectual disability (Chahrour and Zoghbi, 2007). The disease is caused by mutations in the (*MECP2*) gene encoding the transcription repressor methyl-CpG-binding protein 2 (Amir et al., 1999). As RTT patients and genetic mouse models exhibit dendritic abnormalities such as decreased spine density (reviewed in Xu et al., 2014), the disease was originally attributed to *MECP2* deficiency in the neurons. However, studies of RTT mouse models have shown that all types of glial cells including microglia ubiquitously express this gene. In mice, the loss of *Mecp2* in microglia leads to the release of high levels of glutamate, resulting in neurotoxicity and dendritic damage *in vitro* (Maezawa and Jin, 2010). Recently, microglia (in *Cx3cr1-GFP* reporter mice) were implicated in RTT through their excessive removal of axon terminals at disease end-stages in *Mecp2* null mice (Schafer et al., 2016). However, this process was independent from microglial loss of *Mecp2* expression, suggesting their contribution to the pathological “de-wiring” through the engulfment of synaptic elements rendered vulnerable by the loss of *Mecp2* in neurons or other glial cells (Schafer et al., 2016).

*Autism spectrum disorders (ASDs)* are characterized by impaired social communication as well as restrictive and repetitive patterns of interest and behaviors. ASDs are diagnosed at 2–3 years of age, often with clinical signs visible earlier. A common anatomical endophenotype is the transient brain overgrowth measured between 2 and 6 years of age that normalizes during adolescence or adulthood (reviewed in Courchesne et al., 2007, 2011). The cause is unknown, but may reflect abnormal axonal sprouting, cell proliferation, or deficient removal of neurons, synapses or glial cells. Impairment in the processing and integration of multiple sensory and emotional inputs, characteristic of ASD, was proposed to result from connectivity defects, notably related to dysregulated neurogenesis and neuronal migration (Packer, 2016). The connectivity hypothesis is consistent with the local variations of spine density, e.g., increased among cortical layers (Hutsler and Zhang, 2010), and altered excitation/inhibition ratio observed in sensory, social or emotional brain regions of ASD patients (Rubenstein and Merzenich, 2003). Moreover, among the hundreds of genes associated with ASD by genome-wide association studies and whole genome sequencing (Betancur, 2011; Pinto et al., 2014; Yuen et al., 2017), several were found to be involved in synapse assembly and maintenance (Peca and Feng, 2012; Pinto et al., 2014; Yuen et al., 2017). Postmortem transcriptional analyses of ASD brain samples additionally showed a down-regulation of expression modules enriched with genes related to synaptic transmission (Voineagu et al., 2011; Gupta et al., 2014).

Considering the physiological role of microglia in neurogenesis control, circuit wiring, as well as synapse stabilization and pruning, their function in ASD has been investigated (reviewed in Koyama and Ikegaya, 2015; Edmonson et al., 2016). One of the first postmortem studies of ASD patients reported an increased immunoreactivity for MHC class II (HLA-DR) within the cerebellum and cerebral cortical regions (Vargas et al., 2005). Since then, other postmortem studies confirmed that microglial alterations, mainly related to cellular density, soma volume and complexity of ramifications, were more frequent in ASD patients than age-matched controls (Morgan et al., 2010; Lee et al., 2017). However, not all ASD cases exhibited microglial abnormalities (Morgan et al., 2010), which may reflect a diversity of etiologies. Increased binding for translocator protein (TSPO), notably expressed by microglia and induced in response to inflammatory stimuli (Rupprecht et al., 2010; Karlstetter et al., 2014; Sandiego et al., 2015), was also measured by positron emission tomography (PET) imaging in several brain regions of ASD patients (Fatemi et al., 2012; Suzuki et al., 2013). Dysregulation of microglia in ASD is supported by postmortem transcriptional analyses showing an upregulation of gene expression modules enriched with microglial markers (Voineagu et al., 2011), and genes associated with an anti-inflammatory state and the anti-viral type-I interferon pathway (Gupta et al., 2014). Rare genetic variants of *CX3CR1* were also associated with an increased risk of ASD (Ishizuka et al., 2017). *Cx3cr1* knockout mice displayed phenotypes reminiscent of autism such as repetitive behavior and social interaction defects that could be caused by abnormal connectivity (Paolicelli et al., 2011; Zhan et al., 2014). In these mice, the survival of cortical neurons was impaired (Ueno et al., 2013), hippocampal excitatory synapses showed morphological and physiological features of immaturity (Paolicelli et al., 2011; Rogers et al., 2011; Zhan et al., 2014), the maturation of glutamatergic thalamocortical synapses was delayed during postnatal development (Hoshiko et al., 2012), while the positioning of neocortical interneurons was altered prenatally (Squarzoni et al., 2014). These defects could result from a delayed microglial colonization of specific brain regions including the hippocampus and cerebral cortex during development (Paolicelli et al., 2011; Hoshiko et al., 2012; reviewed in Paolicelli et al., 2014). Overall, these findings indicate that environmental risk factors, particularly perinatal infection, could impair the crucial synaptic pruning function of microglia (Knuesel et al., 2014; Delpech et al., 2016; Mattei et al., 2017). The prevalence of microglia with a primed morphology that was observed in a subset of young (6-year-old) ASD patients (Morgan et al., 2010) support this hypothesis of an active, causative role of microglia, but direct evidence is lacking. Microglial priming might alternatively reflect a secondary reaction to neuronal apoptosis and circuit rewiring that occurred to compensate for an early brain overgrowth or increase in spine density.

*Obsessive-compulsive disorder* (OCD) is characterized by recurrent and uncontrollable thoughts (obsessions) and actions (compulsions) leading to socially-invalidating behaviors such as stereotypy, trichotillomania and excessive cleaning. It is considered a heterogeneous disorder with distinct subtypes having different etiologies (Hirschtritt et al., 2017). Based on a knockout mouse model for the homeobox gene *Hoxb8*, expressed in the myeloid lineage, which showed compulsive grooming (Chen et al., 2010), microglial genetic deficiency was proposed to induce OCD. It was also reported that patients with frontotemporal dementia that carried mutations of the gene encoding progranulin (*GRN*), as well as mice deficient for this gene, displayed OCD and self-grooming behavior, respectively (Lui et al., 2016; Krabbe et al., 2017). While *Grn* function and expression pattern remain poorly-defined, microglia from *Grn* knockout mice, when co-cultured with neurons, more actively internalized synaptophysin-positive puncta that co-labeled with C1qa, correlating with a selective loss of inhibitory synapses in the ventral thalamus (Lui et al., 2016; Krabbe et al., 2017). This loss could contribute to the thalamic hyperexcitability measured in these mice (Lui et al., 2016; Krabbe et al., 2017), which is reminiscent of the dysfunctional striato-thalamo-cortical circuits described in OCD patients (Burguière et al., 2015). Crossing the *Grn* knockout mice with *C1qa* knockout mice prevented the excessive synaptic pruning and rescued the OCD phenotype (Lui et al., 2016). Microglia-specific *Grn* knockouts similarly displayed an excessive grooming phenotype at adulthood (Krabbe et al., 2017), further implicating microglia in this disorder.

## Roles of Microglia in Adult Neuropsychiatric Diseases

Genome-wide studies revealed an association of immune, neuronal and synaptic pathways with several adult neuropsychiatric diseases (The Network and Pathway Analysis Subgroup of the Psychiatric Genomics Consortium, 2015), suggesting that the inflammatory CNS milieu and microglia are implicated in these disorders, either in pathogenesis or progression. In this section, we review the pre- and clinical evidence that altered microglial physiological functions may contribute to psychiatric disorders with an onset in late adolescence or adulthood (Figures 1, 2).

*Alcohol and drug abuse* result in cognitive impairment and neurodegeneration. The proposed mechanisms, mainly derived from animal studies, include signaling through microglial Toll-like receptors (TLR), which form a subfamily of pattern recognition receptors (PRRs) allowing innate immune cells to detect changes in homeostasis through the recognition of an array of pathogen-associated molecular patterns (PAMPs; linked to microbial pathogens and cellular stress) and danger-associated molecular patterns (DAMPs; released by cellular damage). The recruitment of TLRs contributes to amplifying microglial release of pro-inflammatory mediators (Stridh et al., 2013; Yao et al., 2013).

A first binge of ethanol in rats induced microglial priming leading to increased immunoreactivity for CR3 and IBA1, and enhanced hippocampal levels of TNFα (tumor necrosis factor alpha), upon a subsequent intake (Marshall et al., 2016). Alcohol exposure in mice similarly potentiated the effects of LPS on brain levels of IL-1β and TNFα, impaired hippocampal neurogenesis, and increased immunoreactivity against IBA1 (Qin et al., 2008). After an acute binge of alcohol in mice, microglial depletion by treatment with a CSF1R inhibitor (PLX5622) increased the brain levels of anti-inflammatory genes, while reducing pro-inflammatory ones (e.g., *Tnfa*, *Ccl2*, Walter and Crews, 2017). Supporting TLRs involvement in microglial pro-inflammatory state upon alcohol exposure, administration of the TLR3 agonist Poly I:C before alcohol enhanced brain levels of TNFα, IL-1β, IL-6 and MCP-1 (monocyte chemoattractant protein-1) mRNA and protein, immunoreactivity against IBA1 and NOXgp91phox (a subunit of NADPH oxidase that generates superoxide and is expressed mainly by microglia), as well as neurodegeneration in the cerebral cortex and hippocampus of mice (Qin and Crews, 2012). These effects were blocked by treatment with minocycline, a tetracycline antibiotic with neuroprotective and anti-inflammatory properties, or the opioid receptor antagonist naltrexone, which also exerts anti-inflammatory effects notably via binding to TLRs (Qin and Crews, 2012). Knockdown of TLR4, which is triggered by LPS and recognizes DAMPs released by injured tissue, prevented alcohol-induced increase of IBA1-immunoreactivity and protected against neuronal apoptosis in cerebral cortex of mice (Alfonso-Loeches et al., 2010). Alcohol-preferring rats had high levels of TLR4 protein and MCP-1 in the central amygdala (CeA) and ventral tegmental area (VTA), while inhibition of both proteins in these areas decreased the excessive alcohol intake, suggesting that TLR4/MCP1 signaling might regulate alcohol self-administration (June et al., 2015). In human, the levels of microglia-associated MCP-1 were increased in postmortem VTA, CeA, substantia nigra, and hippocampus of alcoholics compared to healthy controls (He and Crews, 2008). An increased mRNA expression of *TLR7*, which is activated by single-stranded RNA, and *ITGAM* (encoding CD11b), was also measured in postmortem hippocampus of alcoholics (Coleman et al., 2017). These overall findings present microglial TLRs as promising therapeutic targets for alcoholism.

Similarly, TLRs have a critical contribution to opioids dependence, which is associated with cognitive deficits during both abuse and withdrawal periods, affecting attention, working and episodic memory, as well as executive functions (Dhingra et al., 2015). Chronic exposure to morphine induced apoptosis of primary fetal human microglia, which could be reversed by the opioid receptor antagonist naloxone, suggesting a prominent role of opioid receptor signaling in this process (Hu et al., 2002). Morphine also led to increased mRNA expression of *Tlr9*, a detector of unmethylated CpG dinucleotides found in bacterial and viral DNA, resulting in the apoptosis of primary mouse microglia (He et al., 2011). Morphine tolerance was postponed by blocking release of pro-inflammatory mediators. Systemic treatment with the phosphodiesterase inhibitor Ibudilast (suppressor of microglial pro-inflammatory response through TLR4 signaling) or minocycline both reduced opioids withdrawal in addition to promoting analgesia (Hutchinson et al., 2009). Microglial pannexin-1 was additionally identified as a potential clinical target for opioids withdrawal. Genetic deletion of microglial *pannexin-1* in mice dampened ATP release from spinal cord dorsal horn microglia, and blunted morphine-induced long-term facilitation, thus reducing the severity of withdrawal without affecting analgesia (Burma et al., 2017).

Methamphetamine causes neuropathology through mechanisms that comprise neurotoxicity to serotonin and dopamine neurons, as well as release of pro-inflammatory mediators, eventually resulting in cognitive impairment (Gonçalves et al., 2010; Xu et al., 2017). Microglial response to inflammatory stimuli measured by PET with TSPO ligands was most pronounced in the midbrain, striatum, thalamus, and orbitofrontal, and insular cortices of human abusers (Sekine et al., 2008). Similarly, the density of IBA1-positive microglia increased in striatum of methamphetamine-exposed mice (Thomas et al., 2004; Lloyd et al., 2017). Acute methamphetamine enhanced mRNA levels of *Tnfa*, *Il6* and *Il1b*, in striatum and hippocampus of mice (Gonçalves et al., 2008). Co-localization of IBA1-positive microglia with the purinergic receptor P2X7R was additionally shown to increase in striatum of exposed mice, while pharmacological blockade or silencing of P2X7R in embryonic stem cell-derived microglia prevented their increased migration, reduced phagocytosis, and enhanced pro-inflammatory release induced by methamphetamine (Fernandes et al., 2016). These findings suggest that modulating microglial phenotype might help to prevent the neurological effects of chronic methamphetamine. Pharmacological treatment with minocycline indeed prevented the reduction of serotonin and dopamine levels, and the behavioral impairment of mice receiving methamphetamine (Zhang et al., 2006). In healthy humans, minocycline similarly decreased the subjective rewarding effects of dextroamphetamine (enantiomer of methamphetamine; Sofuoglu et al., 2011). Likewise, in methamphetamine abusers, Ibudilast reduced the rewarding effects of methamphetamine (Worley et al., 2016).

As with opioids and methamphetamine, the rewarding properties of cocaine are related to an increased release of dopamine in the NAc (Pontieri et al., 1995). Cocaine was shown to interact with microglial TLR4, increase *Il1b* mRNA levels in VTA, extracellular dopamine in NAc, as well as increase conditioned place preference and self-administration (Northcutt et al., 2015). These effects were all suppressed by treatment with TLR4 antagonists in mice, indicating a crucial role of TLR4 in cocaine reward and reinforcement (Northcutt et al., 2015). Increase in IL-1β mRNA and protein levels after acute cocaine administration was also measured in the cerebral cortex and NAc of rats (Cearley et al., 2011). Repeated intake of cocaine was shown to increase TNFα mRNA and protein levels in the NAc, resulting in synaptic depression and suppressed cocaine-induced behavioral sensitization in mice (Lewitus et al., 2016). In this study, microglia displayed increased IBA1-immunoreactivity, enlarged soma, and reduced process arborization in the NAc, and were identified as the cell type responsible for the release of TNFα using microglia-specific knockouts. Dopamine was additionally shown to increase microglial release of TNFα *ex vivo*, through the recruitment of D2 dopamine receptors (Lewitus et al., 2016). The weak TLR4 agonist monophosphoryl lipid A (MPLA; variant of LPS) also resulted in the suppression of behavioral sensitization, a process that required microglial TNFα (Lewitus et al., 2016), further supporting the idea that TLRs could be promising therapeutic targets.

*Major depressive disorder* (MDD) is characterized by anhedonia (sense of worthlessness) and cognitive impairment (Krishnan and Nestler, 2008). It affects 10%–15% of the general population worldwide. Functional magnetic resonance imaging (fMRI) and morphometric analysis indicate a consistent reduction in activity and size of the prefrontal cortex in MDD patients (Drevets et al., 1997; Rajkowska et al., 1999). Correlative fMRI analysis revealed an altered connectivity, within and between numerous brain regions relevant to resting mode, cognitive functions, and emotions (reviewed in Mulders et al., 2015). The hypothesis of a decreased connectivity in the prefrontal cortex is supported by reduced spine density and down-regulation of genes related to synaptic function (Kang et al., 2012). Despite the large number of MDD patients, we still have very limited understanding of the pathogenic mechanisms, which are obviously heterogeneous. For instance, a comprehensive comparison of transcription profiles in MDD patients and a mouse model of chronic unpredictable stress identified connectivity modules that were differentially enriched in microglia from each sex (Labonté et al., 2017).

An immunological hypothesis was proposed from the evidence that several core symptoms of MDD resemble sickness behavior (i.e., a set of adaptive behavioral changes comprising lethargy, depressed mood, reduced social exploration and loss of appetite) resulting from infectious or inflammatory conditions (Dantzer et al., 2008). According to this model, the chronicity of inflammation would induce a long-lasting depressive phenotype in subjects that are genetically predisposed or exposed to an adverse environment (Dantzer et al., 2008). This hypothesis is supported by the observation that subsets of MDD patients have elevated levels of circulating cytokines (mainly TNFα and IL-6; Dowlati et al., 2010), and increased expression of innate immunity-related genes in blood (Leday et al., 2018). In the brain, PET studies have reported increased TSPO binding in prefrontal cortex, insula and anterior cingulate cortex of MDD patients that positively correlated with their depression severity (Setiawan et al., 2015). This association of elevated inflammatory status in the CNS with depression severity was particularly significant in patients with suicidal thoughts (Holmes et al., 2018). Consistently, an increased density and enlargement of primed IBA1-positive microglia, associated with the upregulation of the genes encoding IBA1 and MCP-1, was observed in postmortem white matter of dorsal prefrontal and anterior cingulate cortex of depressed patients that had committed suicide (Steiner et al., 2008; Torres-Platas et al., 2014). Preliminary studies using minocycline as an add-on treatment (to selective serotonin reuptake inhibitors) for MDD also brought encouraging results (Dean et al., 2017; Husain et al., 2017) but require replication and further analysis regarding the inflammatory status of the patients.

**Figure 3:**
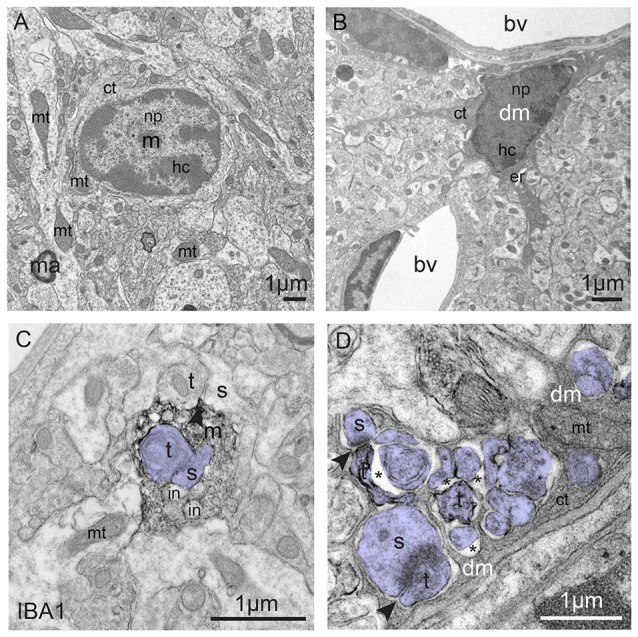
Typical vs. dark microglia. **(A)** Typical microglia (m) that displays a light cytoplasm (ct) and nucleoplasm (np) with a clearly defined heterochromatin (hc) pattern, as well as intact organelles in the hypothalamus of a healthy adult mouse. Mitochondrion = mt; ma = myelinated axon. **(B)** Dark microglia (dm) in the hippocampus of a chronically-stressed *Cx3cr1* knockout mouse showing various signs of oxidative stress, including the darkening/condensation of its cytoplasm and nucleoplasm, making it appear as dark as mitochondria, and dilation of its endoplasmic reticulum (er) bv, blood vessel. **(C)** Processes from typical microglia are generally bulky and make focal contacts with synaptic elements. Several phagocytic inclusions (in) are shown in an IBA1-stained process, in addition to a synapse between an axon terminal (t) and a dendritic spine (s; colored in purple), within the hippocampus of a healthy adult mouse. **(D)** By contrast, dark microglia’s processes extensively encircle synaptic elements (colored in purple), including shrunken terminals undergoing digestion, which are surrounded by extended extracellular space (asterisk), and entire excitatory synapses, as shown in the hippocampus of a chronically-stressed *Cx3cr1* knockout mouse. Microglial contacts with synaptic clefts are indicated by arrowheads in **(C,D)**. Blood vessels, cells and cellular elements are labeled by the large bold font. Organelles and subcellular compartments are labeled by the smaller font.

Rodent models of chronic stress-induced depression revealed that connectivity (assessed by spine density or dendritic arborization) was overall decreased in prefrontal cortex, like in depressed patients, while an opposite effect was observed in NAc and amygdala (reviewed in Christoffel et al., 2011). Chronic stress also inhibited neurogenesis (reviewed in Kang et al., 2016), and affected microglial density, morphology, and gene expression, with modalities that depended on the nature and duration of the stress paradigm and examined brain region(s) (reviewed in Yirmiya et al., 2015; Calcia et al., 2016; Tian et al., 2017). Microglial hyper-ramification was reported in rat and mouse after forced swim or restraint stress (Hinwood et al., 2012, 2013; Hellwig et al., 2016), whereas repeated social defeat or chronic unpredictable stress de-ramified microglia (Kreisel et al., 2014; Wohleb et al., 2015; Milior et al., 2016; reviewed in Tian et al., 2017). These alterations could mediate vulnerability or resilience to depression. First, a number of microglial genes regulated by stress encode proteins modulating synaptic plasticity and adaptive behaviors (IL-1β, TNFα; reviewed in Delpech et al., 2015). Second, phagocytosis of neuronal and synaptic material by microglia (in *Cx3cr1*-GFP reporter mice) increased in response to chronic stress (Milior et al., 2016; Wohleb et al., 2018). Repeated social defeat or chronic unpredictable stress in mice also induced a strong increase in the density of microglia exhibiting signs of oxidative stress thus appearing “dark” in electron microscopy (Bisht et al., 2016; Figure 3). As dark microglia were shown to interact more extensively with synapses than typical microglia, it is possible that their increased prevalence upon chronic psychological stress is related to a pathological rewiring of the brain.

Signaling between the neuronal fractalkine and its receptor CX3CR1 seems particularly relevant to the stress response (Wolf et al., 2013; Paolicelli et al., 2014; Sheridan et al., 2014). Four studies reported that *Cx3cr1*-deficient mice were resistant to chronic stress exposure (i.e., chronic unpredictable stress, forced swim, or a two-hit model combining maternal separation and chronic unpredictable stress at adulthood (Hellwig et al., 2016; Milior et al., 2016; Rimmerman et al., 2017; Winkler et al., 2017)). Altogether these murine studies suggest that the resistance of microglia to stress-induced changes in density and morphology (Hellwig et al., 2016; Milior et al., 2016) could prevent the detrimental behavioral outcomes of stress. Nevertheless, at the molecular level, microglia from *Cx3cr1* knockout mice responded to stress, albeit differently than microglia from wild-type mice. For instance, chronic stress downregulated *Ptgds* (prostaglandin D2 synthase) and *Gpr88* (a G protein coupled receptor) in microglia from wild-type mice, but upregulated these genes in *Cx3cr1* knockout mice. As treatments modulating microglial functions (e.g., minocycline, GM-CSF and M-CSF, LPS, overexpression of *Il1ra*) were shown to rescue microglial alterations, neurogenesis, and behavioral dysfunctions in stressed animals (reviewed in Hinwood et al., 2013; Kreisel et al., 2014), some of the stress-induced microglial changes might be related to resilience, instead of conferring vulnerability to depression (reviewed in Kreisel et al., 2014; Yirmiya et al., 2015).

*Schizophrenia* is a heterogeneous mental disorder associated with positive (e.g., hallucinations) and negative (e.g., abnormal social behavior, confused thinking) symptoms. It affects 0.3%–3% of the general population worldwide, depending on the inclusion criteria, and emerges in late adolescence or early adulthood (van Os and Kapur, 2009). Schizophrenic patients present an impaired hippocampal neurogenesis (reviewed in Kang et al., 2016) and altered neuronal connectivity, illustrated by a reproducibly low spine density in several regions of the cerebral cortex and hippocampus, which altogether contribute to gray matter loss, reduced hippocampal size, and functional hypoactivity (reviewed in Penzes et al., 2011). Moreover, a decrease of inhibitory markers (GAD67, the GABA synthetizing enzyme and parvalbumin), both at mRNA and protein levels, support the hypothesis of an excitation/inhibition imbalance (reviewed in Gonzalez-Burgos et al., 2015; Canitano and Pallagrosi, 2017).

Indirect evidence that microglia could be implicated in schizophrenia came from clinical studies reporting a significant decrease of positive and negative symptoms when minocycline was added to the antipsychotic treatment (Miyaoka et al., 2008, 2012; Levkovitz et al., 2010). Large-scale studies unraveled the risk association of schizophrenia with genetic markers across the MHC locus, particularly genes encoding complement C4, proposing an immune vulnerability that could involve microglia (Sekar et al., 2016). C4 expression was detected in subsets of neurons and astrocytes from human postmortem hippocampus and prefrontal cortex, while *C4* knockout mice displayed impaired synaptic refinement (Sekar et al., 2016). Moreover, addition of C4 increased the phagocytosis of synaptosomes by human iPSC-derived microglia *in vitro* (Sellgren et al., 2017). Whether the reduced spine density reported in postmortem schizophrenia brains resulted from an impaired synaptogenesis or an exacerbated microglial phagocytosis is still undetermined due to the lack of relevant human data. In addition, PET revealed an increased binding of TSPO ligands in the hippocampus (Doorduin et al., 2009), total gray matter (van Berckel et al., 2008), and gray matter of frontal and temporal lobes of schizophrenic patients compared to healthy controls (Bloomfield et al., 2016). However, further investigations using more selective TSPO ligands are necessary for conclusive findings (Coughlin et al., 2016; Notter et al., 2017). In postmortem prefrontal cortex, genes of a specific inflammatory module comprising *IL6*, *IL8*, and *SERPIN3* were shown to be overexpressed, while the density of MHC class II-positive microglia was increased (Fillman et al., 2013). In these samples, dystrophy of MHC class II-positive microglia, revealed by the thinning, shortening and fragmentation of their processes, was additionally observed (Radewicz et al., 2000; Wierzba-Bobrowicz et al., 2004, 2005; Busse et al., 2012). Overall, an alteration of the genetic inflammatory profile in prefrontal cortex was fourfold more frequent in schizophrenic vs. control patients (Fillman et al., 2013). Nevertheless, changes of microglia in schizophrenia may depend on the different etiologies, disease progression, as well as history of treatment and substance abuse.

*Bipolar disorder* (BD) is characterized by recurrent episodes of mania followed by depression, generally beginning in adolescence or early adulthood. Few studies have investigated the roles of CNS immune regulation in BD specifically. A potential role of the immune system was suggested by the increased plasmatic levels of pro-inflammatory cytokines measured in patients during acute episodes of mania or depression, compared to recovery phases (Muneer, 2016a). Postmortem analyses reported increased mRNA and proteins levels of IL-1β, iNOS and CD11b in frontal cortex of BD patients (Rao et al., 2010). Exacerbated inflammatory response was also indicated by PET imaging showing a significant increase of TSPO binding in the right hippocampus of BD patients (Haarman et al., 2014). Although BD has a high heritability, its genetic determinants are unknown, and there is no animal model that may reliably distinguish BD from unipolar depression or schizophrenia. Nevertheless, one specific trait of BD seems to be the decrease in BDNF serum levels during manic or depressive phases. This decrease in BDNF correlated with the clinical severity, normalizing at recovery phases or in medication-induced remissions (Lin, 2009; reviewed in Muneer, 2016b). Whether microglial-derived BDNF linked to dendritic spine formation in mouse (Parkhurst et al., 2013) could underlie different synaptic plasticity levels between acute and remission episodes remains unclear. Although highly speculative, this mechanism could possibly account for the maladaptive behavior of BD patients.

### Eating Disorders

Very little data exist on the pathogenesis of eating disorders, such as anorexia nervosa and bulimia nervosa, with relation to microglia. One study showed that stimulation of TLR2 by intracerebroventricular injection of the synthetic ligand Pam3CSK4 induced anorexia and increased IBA1-positive microglial density and structural contacts with proopiomelanocortin (POMC) neurons in the hypothalamic arcuate nucleus of mice (Jin et al., 2016). However, further experiments are required to determine whether this anorexia mediated by TLR2, which is likely a transient symptom of sickness behavior, shares pathogenic mechanisms with anorexia nervosa, which develops over months to years in humans. Microglia were also proposed to mediate the excessive dietary intake, by acting on the hypothalamic control of energy balance (Valdearcos et al., 2014, 2017). In particular, silencing microglial inflammatory signaling via NF-kB pathway (using microglia-specific knockouts for *Ikkβ*) or depleting microglia (with the CSF1R inhibitor PLX5622) protected high fat diet-fed mice against hyperphagia (Valdearcos et al., 2014, 2017). In contrast, microglia-specific knockouts of A20, a primary negative regulator of NF-kB activity, showed exaggerated pro-inflammatory microglial activities *ex vivo*, changes in hypothalamic densities of microglia and infiltrating myeloid cells, as well as metabolic dysfunction and obesity, independently from the diet (Valdearcos et al., 2017). These findings open the possibility to exploit microglial inhibitors in the context of human metabolic pathologies.

### Sleep Disorders

Sleep is vital to maintaining health. It allows to restore synaptic homeostasis, clear the brain from toxins, and consolidate memory (Diekelmann and Born, 2010; Xie et al., 2013; Tononi and Cirelli, 2014). Sleep comprises a non-rapid-eye-movement state identified by slow EEG waves and a rapid-eye-movement sleep (REM) state associated with brain activation, as well as inhibition of muscle tone and saccadic eye movements. A PET longitudinal follow-up study showed that patients affected by idiopathic REM sleep behavior disorder were more vulnerable to subsequently developing PD and other synucleinopathies, i.e., neurodegenerative diseases characterized by an abnormal accumulation of α-synuclein in neurons and glial cells. These patients displayed increased TSPO binding in the substantia nigra, associated with a decreased dopaminergic activity in the putamen (Stokholm et al., 2017), suggesting that immune cell activation could represent a biomarker and/or therapeutic target for both idiopathic REM sleep behavior disorder and synucleinopathies. Microglia are linked to several sleep disorders, including sleep deprivation discussed below (reviewed in Nadjar et al., 2017).

Sleep deprivation induces tiredness, sleepiness, irritability and concentration difficulties, as well as more serious outcomes like cognitive impairment, anxiety and neurodegeneration when it becomes chronic (Musiek and Holtzman, 2016; Pires et al., 2016). Studies in rodent models of chronic sleep deprivation reported an increased expression of CR3 protein and hypertrophy of CR3-positive microglia in rat hippocampus (Hsu et al., 2003) and enhanced mRNA levels of pro-inflammatory cytokines in mouse brain (Wisor et al., 2011). Sleep deprived mice also presented an increase in EEG slow waves that could be reversed by the administration of minocycline (Wisor et al., 2011). An increase in IBA1-positive microglial co-localization with VGLUT1-positive glutamatergic terminals, associated with less ramified morphologies, was reported in mouse prefrontal cortex upon chronic sleep deprivation (Bellesi et al., 2017). Since the brain levels of C3 protein were concomitantly enhanced (Bellesi et al., 2017), the authors proposed that complement-mediated synaptic pruning might be exacerbated by chronic sleep deprivation. The expression of MER, regulating microglial process motility and phagocytosis, was also increased by chronic sleep deprivation (Bellesi et al., 2017). Whether microglia help to restore the disrupted homeostasis during chronic sleep deprivation, or contribute to its detrimental consequences on synaptic loss and cognitive dysfunction remains to be investigated.

## Defective Microglial Wiring of the CNS in Old Age

Microglia are not exempted from the natural transformation experienced by the body over time (Tay et al., 2017b). Here we discuss the impact of age-related microglial alterations and their consequences on cognitive functions (Figures 1, 2).

### Physiological Aging

One of the first noticeable changes with aging was the increased prevalence of dystrophic microglia primarily identified by their cytoplasmic fragmentation and appearance of short, twisted processes (Streit et al., 2004; Ritzel et al., 2015). Although seen in young age (Streit et al., 2004), their increase could imply a reduced area surveyed for harmful debris. Additionally, ameboid microglia, also encountered in early CNS development (Kaur and Ling, 1991; Leong and Ling, 1992), became more prevalent with aging (Rozovsky et al., 1998; Jyothi et al., 2015). Aged microglia, in general, showed increased oxidative stress, corroborated *ex vivo* by their increased production of reactive oxygen species (ROS; Ritzel et al., 2015) and reduced antioxidant glutathione activity (Njie et al., 2012). Dark microglia displaying various signs of oxidative stress and encircling synaptic elements with their highly-ramified processes showing strong immunoreactivity for CD11b (Bisht et al., 2016), a component of CR3 that is involved in synaptic pruning, also become numerous with aging. This suggests that dark microglia could mediate synaptic loss and ultimately lead to cognitive dysfunction (Figure 3).

In aging, there is also an increased presence of reactive microglia showing increased MHC class II-immunoreactivity, enlarged cell bodies, and reduced number of short and thick processes (Perry et al., 1993; Rozovsky et al., 1998; Hefendehl et al., 2014). As for the dystrophic cells described above, shorter microglial processes could be detrimental to their ability to survey the parenchyma. A reduced area surveyed by individual microglia was indeed measured in aging, but found to be compensated by a 14% increase in microglial cells in the somatosensory cortex of *Cx3cr1*-GFP reporter mice *in vivo* (Hefendehl et al., 2014). An increased microglial density is supported by previous murine studies in the retina (*Cx3cr1*-GFP mice; Damani et al., 2011), dentate gyrus and hippocampus CA1 (CR3-immnostaining; Mouton et al., 2002), as well as auditory and visual cortices (IBA1-immunostaining; Tremblay et al., 2012). Remarkably, the disparity where females had greater numbers of CR3-microglia in the dentate gyrus and CA1 than males became more pronounced with aging (Mouton et al., 2002). Another striking change in old rodents is the abundance of microglial clusters, seen in white matter (CR3-staining; Perry et al., 1993) and cerebral cortex (IBA1-staining, *Cx3cr1*-GFP mice; Tremblay et al., 2012; Hefendehl et al., 2014). The clumping of microglia could arise from their proliferation, or an increased egress from their designated territory (Hefendehl et al., 2014), resulting in parenchymal areas devoid of surveillance, which could make the brain more vulnerable to the harmful accumulation of debris. In addition, a decrease in microglial process motility during aging was detected in mouse cerebral cortex *in vivo* (Hefendehl et al., 2014) and retina *ex vivo* (Damani et al., 2011). Genes related to process motility like *Pf4*, *Itga4* and *Cxcr4* were indeed measured at lower levels in aged vs. young mouse cerebral cortex (Orre et al., 2014). These aged-related alterations of microglial density, distribution, morphology and dynamics altogether suggest a decline in their capacity to properly survey and preserve the brain milieu against threats.

Microglial release of pro- and anti-inflammatory cytokines becomes disturbed as well with age. Increased brain levels of TNFα and IL-1β mRNA or proteins were measured in aged rodents in steady-state conditions or upon immune challenge (Sierra et al., 2007; Stichel and Luebbert, 2007; Njie et al., 2012). Previous *in vitro* studies have shown that TNFα mediates neuronal loss (De Lella Ezcurra et al., 2010; Kaur et al., 2014), notably through microglial “phagoptosis” of viable neurons (Neniskyte et al., 2014). IL-1β has the capacity to alter microglial morphology from ramified to ameboid in rat hippocampal slice culture (Hailer et al., 2005). IL-1β by itself did not affect neuronal viability but its inhibition using IL-1ra, a receptor antagonist of IL-1, reduced neuronal damage after an excitotoxicity insult in rat hippocampal slice culture (Hailer et al., 2005). Pro-inflammatory IL-6 mRNA and proteins were also increased in the brain (Sierra et al., 2007) and isolated microglia (Ye and Johnson, 1999) from aged mice. Even though IL-6 is expressed by astrocytes, neurons and microglia, only the latter displayed an age-dependent increase in IL-6 production in mice (Ye and Johnson, 1999). In addition, anti-inflammatory cytokines, such as IL-10 and TGFβ1, were detected in larger quantities in the aged brain of mice (Sierra et al., 2007), leading to the hypothesis that anti-inflammatory cytokines increase during aging to dampen the detrimental effects of pro-inflammatory cytokines and prevent inflammation from impairing further microglial functions (Sierra et al., 2007).

Synapses in vulnerable regions are affected over time, as indicated by the age-related decrease in the expression of genes associated with synaptic vesicle trafficking or neuromodulation that was measured in the superior frontal gyrus and postcentral gyrus, and to a lesser degree in the hippocampus and entorhinal cortex, of human postmortem samples (Berchtold et al., 2013). Synaptic loss has been observed in multiple brain regions with aging, for instance the temporal lobe of postmortem human samples (Anderson and Rutledge, 1996), prefrontal cortex of rhesus monkeys (Peters et al., 2008) and olfactory bulb of mice (Richard et al., 2010; extensively reviewed in Petralia et al., 2014). However, in other brain regions, such as the somatosensory cortex of mice, reduced size and long-term stability of spines was instead observed *in vivo* (Mostany et al., 2013). In addition, increased brain levels of C1q, and several other components of the complement pathway, were measured in aged mice and human (Cribbs et al., 2012; Stephan et al., 2013). Mice deficient for C3 were additionally found to be protected from synaptic loss and neuronal death during aging, in the hippocampus CA3, suggesting that microglia are implicated in synaptic loss or remodeling during normal aging by means of the complement pathway (Shi et al., 2015).

### Age-Related Neurodegeneration

Age is the main risk factor for neurodegenerative diseases. The most prevalent, AD and PD, are associated to elevated CNS inflammatory milieu, synaptic dysfunction and loss, cognitive decline and dementia (Šišková and Tremblay, 2013).

AD is characterized by the appearance of neurofibrillary tangles and accumulation of amyloid β (reviewed in Perl, 2010). Seen in multiple brain regions, including frontal cortex (Scheff et al., 1990) and temporal lobes (Scheff and Price, 1993), synaptic loss is an early event in AD (Scheff et al., 2006) that correlates with the severity of cognitive dysfunction (Terry et al., 1991; Spires-Jones and Hyman, 2014). Early onset AD which typically begins around 40 years of age (Seltzer and Sherwin, 1983) has been associated with mutations in *APP*, *PSEN1* and *PSEN2* (Bekris et al., 2010), and also with polymorphisms in several microglial genes including *CD33* (Griciuc et al., 2013; Malik et al., 2013), *ABI3* (Sims et al., 2017), *PLCG2* (Sims et al., 2017), and *TREM2* (Guerreiro et al., 2013; Jonsson et al., 2013; Suárez-Calvet et al., 2016; Sims et al., 2017). Late onset AD is a sporadic form of the disease that affects a majority of patients (up to 95%) and usually develops after 65 years of age (Seltzer and Sherwin, 1983). Chronic psychological stress across the lifespan is considered a main risk factor for this late onset form of AD (Miller and Sadeh, 2014). In mice, exposure to early-life stress was shown to alter the inflammatory response to amyloid β pathology during adulthood (Hoeijmakers et al., 2017). APP/PS1 mice housed with limited bedding and nesting materials in their first postnatal week displayed reduced amyloid β deposition that was notably accompanied by increased CD68- and IBA1-immunoreactivity in the dentate gyrus at 4 months of age (Hoeijmakers et al., 2017).

While the number of microglia is similar between healthy subjects and AD patients (Griciuc et al., 2013), an increased prevalence of reactive microglia positive for MHC class II was described in the cerebral cortex of patients with senile dementia of the AD type (McGeer et al., 1987), both in gray and white matter, particularly in association with the amyloid β plaques (Mattiace et al., 1990). However, an absence of reactive IBA1-positive microglia was also reported in the cortical gray matter of postmortem AD samples (Streit et al., 2009). The authors thus suggested that microglial “activation” could be related to peripheral infections which affect the CNS over the course of AD pathology, rather than the disease itself. Additionally, as in normal aging, an increased prevalence of dystrophic microglia was discerned both in the frontal (Flanary et al., 2007) and temporal (Streit et al., 2009) lobes of postmortem AD brains, even appearing in temporal lobes before Tau pathology (Streit et al., 2009).

Upregulated in plaque-associated microglia (Frank et al., 2008; Melchior et al., 2010; Guerreiro et al., 2013), TREM2 is expressed by dark microglia (Bisht et al., 2016), disease-associated microglia (DAM; Keren-Shaul et al., 2017), and microglia dependent on the TREM2-APOE pathway (Krasemann et al., 2017), three subtypes that were described in AD mouse models. Deletion or dominant negative mutations of *Trem2* were shown to worsen AD progression in the 5xFAD and APP/PS1 mouse models of AD (Jay et al., 2015, 2017; Yuan et al., 2016; Ulland et al., 2017), but *Trem2* deletion also reduced amyloid β burden, increased neuronal loss, prevented microglial association with the plaques, and resulted in their apoptosis in the 5xFAD model (Wang Y. et al., 2015). Genetic deletion of *Trem2* in a mouse model of Tau pathology (PS19) resulted in an attenuated atrophy of the entorhinal and piriform cortices, together with increased protein levels of PSD95 in hippocampus (Leyns et al., 2017). IBA1-positive microglial density and morphology normalized in the piriform cortex and hippocampus, while the levels of genes coding for pro-inflammatory or phagocytic markers (IL-1α, IL-1β, TNFα and C1q) decreased in the piriform cortex (Leyns et al., 2017). IBA1-positive microglia expressing APOE also became less prevalent, within the piriform cortex, indicating a phenotypic shift. By contrast, Tau phosphorylation or solubility were unaltered in the piriform cortex and hippocampus (Leyns et al., 2017). These findings suggest that TREM2 could mitigate microglial response to Tau pathology, thus protecting against neurodegeneration.

Microglial capacity to phagocytose amyloid β efficiently is affected by aging and AD. As a matter of fact, microglia demonstrate an age-dependent ability to phagocytose since microglial cells isolated from 6 to 8 months old wild-type mice could not clear amyloid β fibrils with the same efficiency as microglia isolated at postnatal day 0 (Floden and Combs, 2011). Using acute hippocampal slices from 7 to 9-week old APP/PS1 mice, the phagocytic ability of microglia was also shown to be compromised in amyloid β plaque-burdened areas specifically (Krabbe et al., 2013). Similarly, an age-related decrease in mRNA expression of genes coding for amyloid β receptors (e.g., SRA, CD36, RAGE) or degrading enzymes (e.g., insulysin, neprilysin, MMP9) was detected *ex vivo* in microglia from APP/PS1 mice (Hickman et al., 2008). While amyloid β triggers synaptic loss in rat hippocampal slices (Shankar et al., 2007, 2008), microglial phagocytosis of both amyloid β and dendritic spines was shown to be mediated by microglial TDP-43, a DNA-RNA binding protein encoded by *Tardbp* gene. Microglia-specific knockouts of *Tardbp*, crossed with an APP model, displayed reduced amyloid β load, but also an exacerbated synaptic loss (Paolicelli et al., 2017). For instance, spine density was reduced, while IBA1-microglial co-localization with PSD95 puncta and expression of CD68 were increased in somatosensory and motor cortices (Paolicelli et al., 2017). This finding supports the importance of orienting microglial phagocytosis toward specific cargos in future therapies for AD and other neurodegenerative diseases.

The synaptic impairment caused by amyloid β was also associated with the complement cascade (Hong et al., 2016). Microglia-mediated synaptic pruning was shown to be abnormally activated early in AD pathology, directly affecting synaptic viability (Hong et al., 2016). In particular, an increase of C1q-immunoreactivity was observed in regions vulnerable to synaptic alterations, defined by the loss of synapsin-PSD95 puncta, such as the hippocampus of J20 and APP/PS1 mice. Intraventricular injection of oligomeric amyloid β increased the co-localization of C1q with PSD95, and decreased microglial expression of CD68 in hippocampus. Gene deletion or pharmacological antagonism of C1q in the AD models further halted their loss of synapsin-PSD95 puncta, while rescuing LTP in acute hippocampal slices (Hong et al., 2016). This suggests that oligomeric amyloid β could drive synaptic pruning via the complement cascade or weaken synapses thus leading to their elimination. Additionally, *C3* deficient APP/PS1 mice displayed increased numbers of Vglut2-GluR1 puncta and protein levels of various synaptic markers during aging in the hippocampus, where IBA1-positive microglia recovered surveillant morphologies near plaques (Shi et al., 2017). Brain levels of pro-inflammatory TNFα, IFNγ and IL-12 proteins were also found to be reduced, and those of anti-inflammatory IL-10 to be increased (Shi et al., 2017).

The neuropathology of PD is characterized by a loss of dopaminergic neurons in the midbrain, accompanied by the presence of Lewy Bodies, which are aggregates positive for α-synuclein, and chronic elevation of brain inflammatory responses (reviewed in Wang Q. et al., 2015). Genetic and sporadic forms of the disease were both described (reviewed in Schneider and Obeso, 2015; Poewe et al., 2017). Early onset PD, which primarily affects individuals younger than 40 years of age, is caused by mutations of *SNCA*, *PINK1*, *DJ-1* and *Parkin*, or exposure to neurotoxins (reviewed in Schrag and Schott, 2006). Late onset PD that emerges between 55 and 65 years of age is instead categorized as sporadic, and associated with mutations and variants of *LRRK2* (reviewed in Volta et al., 2015) that is notably, among other cell types, expressed by microglia (reviewed in Russo et al., 2014). Polymorphisms in genes encoding pro-inflammatory cytokines such as TNF-α and IL-1β, and MHC class II were associated with a higher risk of developing PD (Wahner et al., 2007; Hamza et al., 2010). Chronic psychological stress was proposed to confer an increased susceptibility risk to late onset PD (reviewed in Hou et al., 2014; Vyas et al., 2016). In mice, chronic restraint stress that occurs before administration of the neurotoxin 1-methyl-4-phenyl-1,2,3,6-tetrahydropyridine (MPTP), causing the death of midbrain dopaminergic neurons, was shown to exacerbate the motor deficits, learning impairment, further reduce the dopaminergic levels, and increase the loss of dopaminergic neurons in the substantia nigra (Lauretti et al., 2016). In wild-type mice, chronic restraint stress similarly induced a loss of dopaminergic neurons, increased the aggregation of α-synuclein, reduced proteins levels of CX3CR1 and IBA1, as well as IBA1-positive microglial density in the substantia nigra (Ong et al., 2017).

In postmortem PD brain, reactive microglia immunopositive for MHC class II, ICAM-1 (CD54), LFA-1 (CD11a), CD68, TLR2, and displaying ameboid morphologies, were encountered among several regions comprising the substantia nigra, striatum and hippocampus of postmortem PD brains (Imamura et al., 2003; Doorn et al., 2014). Microglial alterations were similarly reported in animal models of PD induced by neurotoxins. For instance, rotenone increased the density of CR3-positive microglia and altered their morphology toward enlarged cells with short, stubby processes, in the striatum and substantia nigra of rats (Sherer et al., 2003). MPTP also increased the density of MHC class II-positive microglia and their heterogeneity, especially in the substantia nigra and globus pallidus, resulting in the concomitant presence of ramified, ameboid, and multinucleated morphologies in monkeys (Hurley et al., 2003). Administration of MPTP in *Ifng* knockout mice reduced the loss of dopaminergic neurons and terminals, as well as decreased CD11b-positive microglial density in the substantia nigra (Mount et al., 2007). *In vitro*, exogenous application of aggregated α-synuclein was sufficient to transform microglia into ameboid cells, increase their phagocytosis of α-synuclein, as well as exacerbate oxidative stress (production of ROS) and the death of dopaminergic neurons in mesencephalic neuron-microglia culture (Zhang et al., 2005). The phagocytosis of α-synuclein is an age-dependent ability, with isolated microglia from older mice showing a reduced capacity to clear α-synuclein *ex vivo* (Bliederhaeuser et al., 2016). In a mouse model of PD that expresses a mutant form of human α-synuclein, IBA1-immunoreactivity was found to be dramatically increased in the spinal cord, where increased mRNA and proteins levels of AXL were also detected, in exclusive association with IBA1-positive cells (Fourgeaud et al., 2016). By contrast, upregulation of AXL was found to be minimal in the brain (Fourgeaud et al., 2016). The authors speculated that wild-type microglia might execute TAM-dependent phagoptosis of the distressed motor neurons, thus prolonging survival, considering that *Thy1-Syn*^hA53Ttg^ mice also knockout for *Mer* and *Axl* displayed a modest life extension (Fourgeaud et al., 2016). However, the roles of microglia in PD pathogenesis remain largely unknown.

## Perspectives

**Figure 4:**
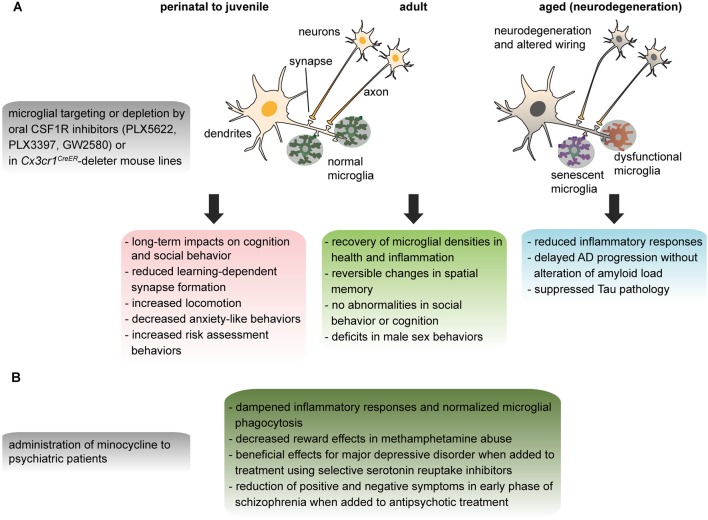
Effects of microglial targeting in animal models and human patients discussed in this review. **(A)** Temporal effects of transient microglial depletion in rodents and nonhuman primates across the lifespan. **(B)** Potential clinical effects of minocycline on modulating the roles of microglia in adult psychiatric diseases.

In recent years, clinical and preclinical studies have advanced our understanding of the roles of microglia in modulating cognitive functions, and their implications in neurodevelopmental, neuropsychiatric, and neurodegenerative disorders. The evidence that CNS inflammatory response is exacerbated in several psychiatric diseases mainly came from gene expression and immunohistochemical postmortem studies, and are now supplemented by PET neuroimaging with TSPO ligands (reviewed in Mondelli et al., 2017). However, current tracers have limitations in specificity and sensitivity. Thus, the development of microglia-specific ligands is a high priority. A selective antagonist of CX_3_CR1, 2-[^18^F]FBTTP, was radiosynthetized and shown to cross the blood-brain barrier using PET imaging in mice. Its application in models of “neuroinflammation” is currently investigated (Mease et al., 2015). In parallel, clinical trials have started to assess the therapeutic potential of the tetracycline derivative, minocycline (Figure 4). Although its mechanisms of action remain elusive, minocycline has neuroprotective properties and is efficient at dampening pro-inflammatory responses and normalizing microglial phagocytosis (Plane et al., 2010; Garrido-Mesa et al., 2013; Mattei et al., 2014). Due to its excellent safety profile, it has been used alone or as an add-on treatment in numerous clinical trials for neurodegenerative diseases, and more recently, in psychiatric disorders, as we have discussed. Current clinical trials now couple the determination of brain inflammatory status (by PET imaging, combined with CSF or blood analysis), to minocycline response (e.g., clinical trial NCT02362529), in order to determine how levels of pro-inflammatory markers before the treatment could predict the clinical response. If the therapeutic effects of minocycline depend on its anti-inflammatory action, one would expect patients with an exacerbated inflammatory profile to be most responsive. Of note, even if minocycline does not target microglia specifically, it could indirectly modulate their physiological functions via astrocytes and vascular endothelial cells. Studies have demonstrated that astrocytes act under the control of reactive microglia in the context of inflammation (Pascual et al., 2012; Habbas et al., 2015; Liddelow et al., 2017).

Animal models, especially microglia-specific gene knockout strategies, have been particularly informative, considering that the readout of microglial pathophysiological alterations is not easily achieved in human subjects. For instance, several groups have taken advantage of the fractalkine receptor to specifically target microglia by crossing the conditional *Cx3cr1^creER^* mouse lines (Goldmann et al., 2013; Parkhurst et al., 2013; Yona et al., 2013) with transgenics carrying genes of interest flanked by *loxP* sites in different disease paradigms (Figure 4). While the *Cx3cr1*^creER^ lines are exceptional tools for dissecting the specific roles of microglia in the brain, long-lived yolk sac-derived non-parenchymal brain macrophages may be partially targeted as collaterals, thus confounding the observations (Goldmann et al., 2016). To date a bona fide microglia-specific marker that labels all microglia, from the post-erythromyeloid progenitor stage up to old age, is still unavailable. Notably, a somatic mutation in the erythromyeloid progenitor of microglia was shown to cause neurodegenerative disease in mouse and human (Mass et al., 2017). However, as mentioned above, *Tmem119* and *Sall1* were shown to be selectively expressed by microglia across different contexts of health and disease (Bennett et al., 2016; Buttgereit et al., 2016). The impact of pharmacologically depleting microglia (Elmore et al., 2014; Torres et al., 2016; VanRyzin et al., 2016) was additionally explored in multiple preclinical animal studies (reviewed in Han et al., 2017; Lund et al., 2017; Figure 4). The current evidence suggest that a transient depletion during early postnatal and juvenile stages has a prolonged impact on cognition and social behavior (Parkhurst et al., 2013; VanRyzin et al., 2016), in contrast to the reversible alterations induced by adult treatment with the selective CSF1R inhibitor PLX3397 (Elmore et al., 2014; Torres et al., 2016). Two independent investigations in AD mouse models, APP/PS1 (Olmos-Alonso et al., 2016) and 5xFAD (Spangenberg et al., 2016), further inhibited microglial proliferation or depletion using the CSF1R inhibitors GW2580 and PLX3397, respectively. Both studies concluded that the progression of AD pathology was delayed with an overall reduction of pro-inflammatory responses although amyloid β load remained unchanged. In contrast, a study that analyzed in mice the propagation of Tau protein revealed a suppression of Tau pathology when microglia were depleted using PLX3397 or intracerebroventricular infusion of clodronate liposomes, in a viral-driven neuron-specific expression of mutant human *Tau* transgene (Asai et al., 2015). Recently PLX3397 was also administered to two Rhesus macaques, in what is possibly the first attempt to deplete microglia in nonhuman primates, in order to understand the dynamics of their depletion in relation to CNS immune cell activation by PET imaging (Hillmer et al., 2017). TSPO binding suggested differential recovery of microglia after depletion in healthy vs. LPS-challenged macaques (Hillmer et al., 2017). Since these pharmacological compounds also target non-parenchymal CNS macrophages and other myeloid cells in the periphery, further characterizations of their effects based on brain compartments, gray vs. white matter, nature and stage of pathologies, and sex-dependency (VanRyzin et al., 2016; Labonté et al., 2017) are required.

## Conclusion

Overall, the findings presented in this review indicate that microglia are increasingly implicated in the pathophysiology of various developmental and neurodegenerative psychiatric disorders, even though the exact mechanisms underlying this association are still undetermined. Critical physiological roles of microglia, including their secretion of cytokines and neurotrophins, phagocytosis, and interactions with synapses, regulate normal brain development, function and plasticity. Animal studies have revealed that abnormal neuronal and synaptic densities, impaired circuit wiring, and imbalance of excitation/inhibition, can result from altered neurogenesis or neuronal survival, impaired synaptic pruning, or altered levels of pro- and anti-inflammatory cytokines. These deficits and alterations were commonly reported in psychiatric disorders. In particular, environmental insults such as perinatal infection, early postnatal and chronic stress, were shown to compromise physiological microglial functions and induce permanent changes in the brain with direct consequences on mood and cognition. Furthermore, microglia could be primed by an early immunological challenge, rendering them more susceptible to a subsequent insult and favoring the emergence of psychiatric conditions later in life. Considering the brain region-dependent slow turnover of microglia, the accumulation of repetitive priming events such as infections and stress across a lifetime may further precipitate the age-dependent deterioration of their physiological functions, resulting in the onset of cognitive aging and neurodegenerative disorders. Due to their unique mesodermal origin, microglia are promising therapeutic targets to gain access to the CNS without directly modifying non-myeloid cell types of the CNS. To precisely characterize the microglial phenotypes implicated in different psychiatric disorders, improvement in selectivity of radiotracers for patient examinations is required. Finally, better characterization of the existing pharmacological compounds able to modulate microglial phenotypes may accelerate the implementation of effective therapies for psychiatric disorders.

## Author Contributions

TLT, AR and MET designed the review outline, recruited CB, ID, MKS and MSH, and supervised the overall project. All the authors contributed to the literature search and to the manuscript writing.

## Conflict of Interest Statement

The authors declare that the research was conducted in the absence of any commercial or financial relationships that could be construed as a potential conflict of interest.
